# Lose the Nintendo and thou shall be healed! Restoring vision in malingering

**DOI:** 10.3205/oc000192

**Published:** 2022-02-08

**Authors:** Michel van Lint, Dave Kloeck, Elisabeth H. van Aken

**Affiliations:** 1 Antwerp University Hospital, Department of Ophthalmology Edegem, Belgium; 2 Antwerp University, Faculty of Medicine Wilrijk, Belgium; 3 Ghent University Hospital, Department of Head and Skin, Ghent, Belgium

**Keywords:** malingering, non-organic visual loss

## Abstract

Non-organic visual loss can be hard to prove or explain to the parents of affected children at times. Here, we describe a simple yet effective approach that may help solve both issues by ensuring that the patient refrains from visual stimuli.

## Introduction

Non-organic visual loss is common in children [1], [2]. Its prevalence can be as high as 20–50% in children with visual loss [1], [2]. Malingering represents the consciously faked loss of function despite adequate functionality. This usually involves complete or partial loss of vision in one or both eyes. In children, psychological factors should be considered [1], [3].

There already are several tests on how to recognize malingering. Here we describe an alternative approach to both detecting malingering, and restoring the “lost” vision.

## Case descriptions

### Case 1

A 7-year-old girl presents with loss of vision in her left eye. Her history is unremarkable. PVEP is normal, as are pupillary light reflexes. Malingering is suspected, and the parents are advised to protect their child’s eyes by keeping her away from TV, computers, game consoles, and other screens. After one week, the patient returns with normal vision.

### Case 2

An 8-year-old girl presents with transient visual loss in her left eye. An autoimmune component is considered since she has a history of juvenile dermatomyositis, for which she is given prednisolone and methotrexate. As an optic neuritis is suspected, prednisolone is increased from 30 to 40 mg/d, and methotrexate is replaced by mycophenolate mofetil.

Visual acuity is 10/10 for the right eye and 8/10 for the left eye. Slit lamp examination reveals no signs of uveitis. Eye fundus is unremarkable. PVEP, pattern electroretinogram (PERG), and multifocal ERG (mfERG) are all within normal range. Further observation is suggested. During follow-up, visual acuity in the left eye drops to 6/10, but then spontaneously recovers to 10/10 again. Although unproven, functional loss of vision is considered in the differential diagnosis because electrophysiology was unable to support the symptoms.

After two years, the patient returns with painful loss of vision in the left eye down to hand movements. PVEP and mfERG are again normal. Pupillary reflexes do not demonstrate the presence of an RAPD. Refrainment from electronic devices is recommended, except when necessary for school. Vision improves to 0.16 the day afterwards and returns to 1.2 after two weeks.

### Case 3

This case involved a third opinion for a 15-year-old girl with loss of light perception in her right eye. At the time of presentation, she had received a regimen of intravenous steroids for a third bout of optic neuritis twelve days before (first and second episode seven and three months before, respectively). Work-up was unremarkable and ancillary tests involved MRI of brain and spine, anti-MOG/NMO screening, lumbar puncture, OCT, and PVEP. In contrast to the previous episodes, the corticosteroids were to no avail this time.

Normal OCT findings and the lack of an RAPD made the case suspicious for malingering. Restrainment from electronic screens was suggested, and vision returned after 2–3 days.

After three months, the patient returns with bilateral loss of light perception, and refrainment from electronic devices is therefore unhelpful. It is explained that this type of visual loss is often associated with underlying psychological problems. Psychological support is recommended, but the mother decides to go elsewhere for further opinions. Nonetheless, the patient later on receives the psychological work-up as originally suggested. It turns out that school mates had been teasing her all her life, causing a negative self-image. 

### Case 4

An 8-year-old boy presents with recent visual complaints and hearing loss. The visual fields show a bitemporal visual field loss. Urgent MRI of the chiasm is unremarkable, but no contrast agent was given. After careful consideration, the radiologist deems a repeat MRI with contrast unnecessary. Neurological work-up is normal as well. Multichannel VEP is normal and unable to show reduced activity in the nasal retinal fibers. The audiogram is found to be normal.

Observation shows that the patient can run around with his baby sister of a few months old in a carriage without bumping into objects or walls. Malingering is considered, and it is recommended for him to stay away from electronic screens. One week later, the mother explains his vision had improved the day after. It is suspected that the patient “lost” his visual fields because his new sister received more attention than him.

## Discussion

Malingering is a recurrent issue in ophthalmological practice [1]. The loss of vision can be stressful for both parents and the practitioner for fear of missing an important diagnosis and leaving the child blind in one or both eyes.

Malingering can often be suspected by inconsistencies in the examination, e.g. a lack of relative afferent pupillary defect if only one eye is involved, normal pattern visually evoked potentials (PVEP) if visual acuity is counting fingers or worse, eyes following a moving mirror in front of them despite loss of light perception, preserved stereoscopic vision despite low visual acuity in one eye, as well as other discrepancies in potential further tests [3], [4], [5].

Unfortunately, once malingering is confirmed, the issue of loss of vision often persists. The parents remain concerned and second or third opinions are subsequently requested. The suggestion of underlying psychological issues is often met with anger or incomprehension by the parents. The diagnosis usually involves extensive and expensive work-up, including magnetic resonance imaging, visual fields, and electrophysiological tests for the eye, commonly PVEP. If an optic neuritis is suggested, patients often receive high doses of steroids.

Here, we describe the simple act of giving the patient’s eyes some rest, by ensuring that the patient refrains from electronic screens, except when necessary for school. This intervention is often well accepted by the parents. This is not to be understood as a punishment, but proves to be quite helpful in regaining vision quickly.

This approach helps to reassure the parents and the physician. Additionally, it can help avoid costly examinations and prevent the use of unnecessary systemic medications and their potential side effects.

Once vision has recovered, it is often easier to talk to the parents and explain the possibility of underlying issues. This is an important follow-up subject to further explore, because the faked loss of vision is often a cry for attention. As such, the child is not to be punished, but in need of help [1], [2], [3], [4], [5]. Failure to provide adequate help may result in recurrent visual loss and/or functional loss elsewhere.

## Notes

### Competing interests

The authors declare that they have no competing interests.
